# Investigation of protein-protein interactions and hotspot region on the NSP7-NSP8 binding site in NSP12 of SARS-CoV-2

**DOI:** 10.3389/fmolb.2023.1325588

**Published:** 2024-01-18

**Authors:** José Xavier Lima Neto, Katyanna Sales Bezerra, Emmanuel Duarte Barbosa, Roniel Lima Araujo, Douglas Soares Galvão, Marcelo Leite Lyra, Jonas Ivan Nobre Oliveira, Shopnil Akash, Yousef A. Bin Jardan, Hiba-Allah Nafidi, Mohammed Bourhia, Umberto Laino Fulco

**Affiliations:** ^1^ Department of Biophysics and Pharmacology, Bioscience Center, Federal University of Rio Grande do Norte, Natal, Brazil; ^2^ Applied Physics Department, University of Campinas, Campinas, São Paulo, Brazil; ^3^ Physics Institute, Federal University of Alagoas, Maceió, Brazil; ^4^ Department of Pharmacy, Daffodil International University, Dhaka, Bangladesh; ^5^ Department of Pharmaceutics, College of Pharmacy, King Saud University, Riyadh, Saudi Arabia; ^6^ Department of Food Science, Faculty of Agricultural and Food Sciences, Laval University, Quebec City, QC, Canada; ^7^ Department of Chemistry and Biochemistry, Faculty of Medicine and Pharmacy, Ibn Zohr University, Laayoune, Morocco

**Keywords:** SARS CoV-2, NSP7-NSP8 binding site, drug design, computational biology, and infectious disease

## Abstract

**Background:** The RNA-dependent RNA polymerase (RdRp) complex, essential in viral transcription and replication, is a key target for antiviral therapeutics. The core unit of RdRp comprises the nonstructural protein NSP12, with NSP7 and two copies of NSP8 (NSP81 and NSP82) binding to NSP12 to enhance its affinity for viral RNA and polymerase activity. Notably, the interfaces between these subunits are highly conserved, simplifying the design of molecules that can disrupt their interaction.

**Methods:** We conducted a detailed quantum biochemical analysis to characterize the interactions within the NSP12-NSP7, NSP12-NSP81, and NSP12-NSP82 dimers. Our objective was to ascertain the contribution of individual amino acids to these protein-protein interactions, pinpointing hotspot regions crucial for complex stability.

**Results:** The analysis revealed that the NSP12-NSP81 complex possessed the highest total interaction energy (TIE), with 14 pairs of residues demonstrating significant energetic contributions. In contrast, the NSP12-NSP7 complex exhibited substantial interactions in 8 residue pairs, while the NSP12-NSP82 complex had only one pair showing notable interaction. The study highlighted the importance of hydrogen bonds and π-alkyl interactions in maintaining these complexes. Intriguingly, introducing the RNA sequence with Remdesivir into the complex resulted in negligible alterations in both interaction energy and geometric configuration.

**Conclusion:** Our comprehensive analysis of the RdRp complex at the protein-protein interface provides invaluable insights into interaction dynamics and energetics. These findings can guide the design of small molecules or peptide/peptidomimetic ligands to disrupt these critical interactions, offering a strategic pathway for developing effective antiviral drugs.

## 1 Introduction

After the first report in December 2019, the COVID-19 pandemic has spread around the world, causing more than 767 million confirmed infections and almost 7 million deaths until July 2023 (World Health Organization, 2020). Despite the decrease in deaths after the beginning of the vaccination programs, the continuous mutation of the virus has reduced the protective efficacy of the available vaccines and monoclonal antibodies (mAbs) (Harvey et al., 2021; McCarthy et al., 2021; Cox et al., 2023). Moreover, only a few drugs have shown some degree of efficacy in treating the infection and its complications, with some of them being reported to present severe side effects, resistance, and rebound phenomena (Kobryn et al., 2021; Anderson et al., 2022; Charness et al., 2022; Zhai et al., 2022; Iketani et al., 2023; Moghadasi et al., 2023). Thus, intensive research has been carried out to find efficient therapies for the treatment and prevention of the current and future coronavirus outbreaks.

SARS-CoV-2, the pathogenic agent of the COVID-19 outbreak, is a positive-strand RNA virus belonging to the genus betacoronavirus, whose members also include the Middle East Respiratory Syndrome Coronavirus (MERS-CoV) and Severe Acute Respiratory Syndrome Coronavirus (SARS-CoV) (Aggarwal et al., 2021). Its genome encodes four structural proteins, and a series of 16 nonstructural proteins (NSP1-NSP16) that play important roles in RNA synthesis and processing, contributing to coronavirus survival and virulence power (Yadav et al., 2021; Biswas et al., 2021). Among these, NSP12 is one of the most important proteins required for viral growth, being the core component of the replication and transcription machinery of SARS-CoV-2 through its RNA-dependent RNA polymerase (RdRp) activity (Narayanan et al., 2022; Biswal et al., 2021).

Due to its key role in the viral cycle, several papers have been published aiming to provide alternatives for inhibiting NSP12 function (Elkarhat et al., 2022; Bagabir, 2023; Giannetti et al., 2023; Gu et al., 2023). However, up to now, the FDA only approved the antivirals Molnupiravir and remdesivir targeting NSP12, both acting in different ways, with the last being covalently incorporated into the primer strand of replicating viral RNA, terminating chain elongation (Kabinger et al., 2021). However, the effectiveness of both drugs is disputed (Consortium et al., 2021; Butler et al., 2023), and the development of new drugs that can impair or, at least, reduce the functioning of the RdRp could be helpful.

It has been shown that NSP12 exhibits weak catalytic activity by itself in coronavirus, needing support from NSP7 and NSP8 cofactors to increase its binding to the template-primer RNA and enhance the polymerase activity (Peng et al., 2020; Kirchdoerfer and Ward, 2019). Structural analysis of the NSP12-NSP7-NSP8 super complex showed that it is stable as a 1:1:2 complex, with 2 NSP8 proteins (NSP8_1_ and NSP8_2_), and 1 NSP7 bound to NSP12. The precise role of NSP7 is still unclear, but, because of its affinity for NSP8 and lack of affinity for RNA, it has been suggested that its role may be to off-load RNA from NSP8 (Gu et al., 2023). On the other hand, it has been shown that mutations in NSP8 have also been associated with the altered synthesis in SARS-CoV-2 (Anjum et al., 2022; Reshamwala et al., 2021) observed that the mutations in NSP7 and NSP8 proteins are significantly associated with mutations NSP12, as a possible compensatory effect to maintain its function. Besides, the amino acid sequence of the three proteins and the binding site for the dimer NSP7-NSP8 in NSP12 are conserved across the coronavirus family (Kirchdoerfer and Ward, 2019), while mutations of some interface residues lead to weakened RNA replication activity of the RdRp machinery (Biswal et al., 2021).

In this sense, the impairment of the interaction between the proteins that form the NSP12-NSP7-NSP8 super complex could be a quite useful strategy to have some control over the SARS-CoV-2 infection, and the level of conservation of these proteins within the coronavirus family makes it a potential target not only for the current pandemic but also to future outbreaks. Thus, computational and experimental studies have been proposed to evaluate the interactions among these proteins, looking to understand and impair them (Ruan et al., 2021; Mutlu et al., 2022; Sarma et al., 2022). However, the authors are not aware of another study seeking to evaluate and rank the most relevant residue-residue interactions at a quantum level of theory, as well as investigate if the introduction of the viral RNA plus the remdesivir drug can alter the interaction pattern between these proteins since the active site of the NSP12 polymerase is very close to the NSP12-NSP7-NSP8 binding interface.

Protein-protein interactions (PPIs) play an essential role in regulating biological processes. Therefore, investigating these interactions is a crucial step for targeting the interfaces between proteins and using them in drug discovery (Cukuroglu et al., 2014). Understanding the PPI sites is essential to finding hotspots, i.e., certain residues or regions of the proteins that contribute more to the binding energy than other areas (Scott et al., 2016). Therefore, the efforts of many research groups are aimed at both the identification and the search for ways to modulate these hotspots (Ershov et al., 2022). Among the most commonly used computational methods to study the PPIs, are those based on classical molecular dynamics, alanine-scanning, hybrid quantum mechanics/molecular mechanics (QM/MM), and fully Quantum Mechanics (QM) methods (de Oliveira et al., 2020). The latter is becoming popular for this purpose because it provides a good description of the molecular geometries, (relative) binding affinities, and electronic states in the system with high accuracy. This is mainly due to its natural advantage of modeling polarization and charge transfer explicitly, becoming a suitable tool in all phases of *in silico* drug design (Zhou et al., 2010; Raha et al., 2007).

Unfortunately, the large number of atoms in biological molecules makes the accurate complete quantum mechanical description of the interaction energies for proteins with their ligands very costly, especially considering the interaction between proteins. In the last years, linear scaling approaches have been developed to make biological molecules computationally less expensive (Merz, 2014). The fragment-based ones represent an important class, with several schemes of protein decomposition applied to obtain protein properties, including the molecular fractionation with conjugate caps (MFCC), fragment molecular orbital (FMO), molecular tailoring approach (MTA), and generalized energy-based fragmentation (GEBF) (Ryde and Söderhjelm, 2016). Among these schemes, MFCC has been widely employed to calculate the interaction energy between protein amino acids and ligands, as well as in protein–protein complexes, allowing the investigation of a large number of amino acid residues in a protein possible with a small computational cost and high accuracy (Lima Neto et al., 2015; Lima Neto et al., 2018; Bezerra et al., 2019; Albuquerque et al., 2021; Tavares et al., 2021). Recently, the MFCC scheme was used to study the interaction between the hACE-2 and Spike proteins of SARS-CoV-1, SARS-CoV-2, and hCoV-NL63 (Lima Neto et al., 2022), the SARS-CoV-2 Mpro with synthetic peptides (Amaral et al., 2022), and the human leukocyte antigen (HLA-A2) in complex with tumor-associated antigens based on glycoprotein gp100 (Pereira et al., 2021).

Therefore, this work aims to present a first detailed description of the interaction between NSP12-NSP7, NSP12-NSP8_1_, and NSP12-NSP8_2_ proteins at a quantum mechanical level of calculation. For this purpose, we have employed quantum biochemistry techniques within the density functional theory (DFT) framework and molecular fractionation with conjugate caps scheme to calculate the individual contribution of each amino-acid residue for the protein-protein interface. Besides, the complexes in the *apo* form and the presence of the template-primer RNA with remdesivir were used to search for structural and energetic differences between the interaction patterns, since the entrance of the RNA structure in NSP12 occurs close to the region in which it binds to NSP7 and NSP8.

## 2 Materials and methods

### 2.1 Protein-protein complex data and quantum calculations

The structural data of the SARS-CoV-2 RNA polymerase complex (NSP7, NSP8, and NSP12 proteins) solved in the *apo* form (PDB ID: 6M71; 2.90 Å of resolution) (Gao et al., 2020), and with a 50-base template-primer RNA and remdesivir (PDB ID: 7BV2; 2.50 Å of resolution) (Yin et al., 2020), based on a high-resolution description of expertly validated and curated structures in the crystal environment (Burley et al., 2019), not taking into account any dynamics process. We are aware that considering multiple structures could significantly improve the description of protein-ligand and protein-protein interactions, as shown in Ref (Liu et al., 2015). Nevertheless, the sampling of alternative refinement models through MD simulations may bring about a higher computational cost, particularly for large protein targets. Although higher computational cost is certainly not a reason to expect that computed effects are less relevant, the qualitative behavior of individual interaction energies cannot change considerably, i.e., the most energetically relevant residues could be similar by taking into account single or multiple structures.

**FIGURE 1:**
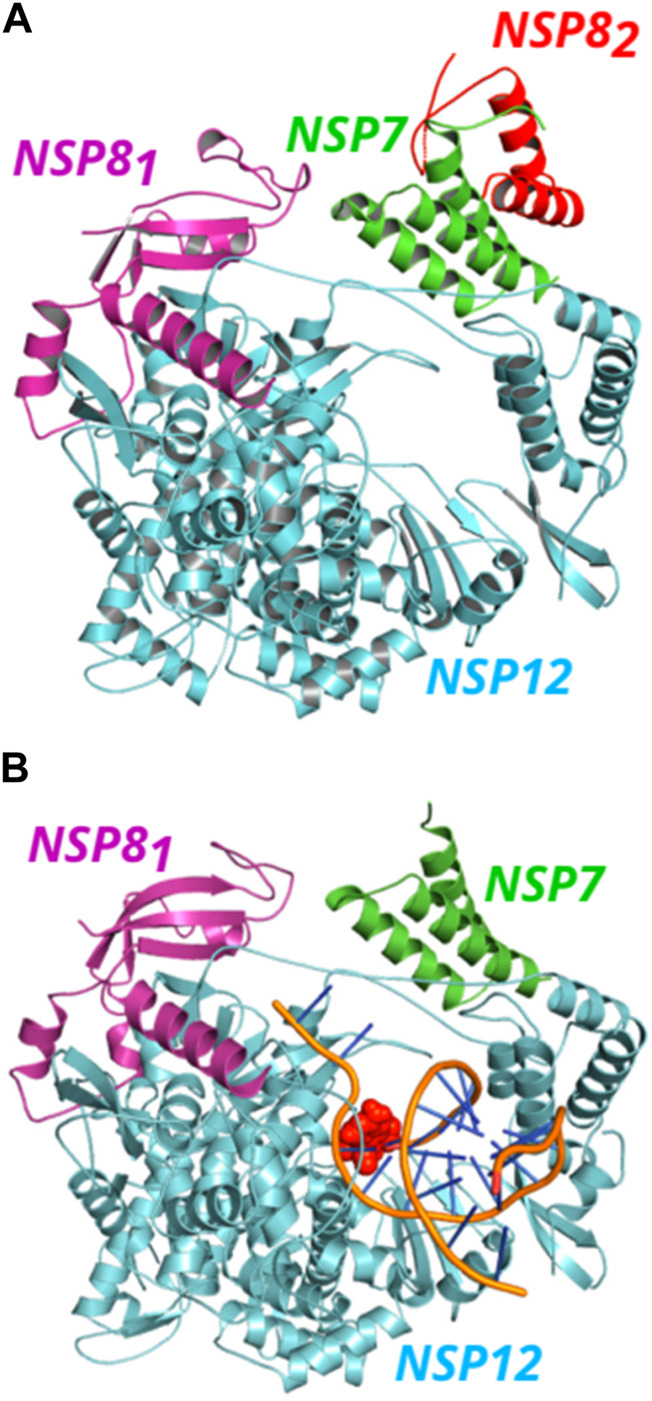
(Color online) Representation of SARS-CoV-2 NSP12 (cyan color) in complex with NSP7 (green color) and NSP8 (magenta and red colors) proteins. **(A)** The NSP81 (magenta color) protein is bound to NSP12, while the NSP82 (red color) also binds to NSP7, forming a dimer in the apo form. **(B)** A view of the NSP12-NSP7-NSP81 structure in complex with the template-primer RNA and remdesivir (orange).

It is important to mention that, in the *apo* form, the RNA polymerase structure from the PDB ID: 6M71 showed one NSP12 protein, one NSP7, and two NSP8 (here termed NSP8_1_ and NSP8_2_), while in the PDB ID: 7BV2, only one structure of each protein is found. See Figures 1A, B to observe the position of each protein in the complexes. NSP12 protein is colored in cyan, while NSP7, NSP8_1,_ and NSP8_2_ proteins are represented in green, magenta, and red, respectively. The position of NSP8_1_ and NSP8_2_ in the complex follows the reference (Gao et al., 2020). In this work, we only took into account the residues present in the crystal structures and some missing residues may have an important impact on the results, mainly the residues at the beginning and end of the NSP7 and NSP8 proteins, that could be in close contact with NSP12 and interact with some of its residues.

We added missing heavy atoms in the proteins, submitted the complexes to the PROPKA 3.1 package (Søndergaard et al., 2011), and included hydrogen atoms in the proteins according to the protonation study, as well as added the hydrogen atoms for the water molecules present in the crystal 7BV2. Afterward, protein main-chain heavy atoms are constrained, and the other atoms are submitted to a classical energy minimization using the Chemistry at Harvard Molecular Mechanics (CHARMm) force field (Momany and Rone, 1992), with the convergence tolerances to 10^−5^ kcal mol^−1^ (total energy variation) and 10^−2^ kcal mol^−1^Å^−1^ (RMS gradient). Since the PROPKA software is slightly sensitive to the ligand pocket geometry, the steps of hydrogen addition/withdrawal and energy minimization are carried out until no difference is observed in the protonation results (Lima Neto et al., 2019).

After the energy minimization step, the dimers NSP12-NSP7, NSP12-NSP8_1_, and NSP12-NSP8_2_ (when they exist) were fragmented following the MFCC scheme (Zhang and Zhang, 2003; da Costa et al., 2012) (see below). The structures generated were submitted to energetic quantum mechanical calculations through the Gaussian (G16) package (Frisch, 2016). The generalized gradient approximation (DFT-GGA) functional B97D (da Costa et al., 2012) was selected to perform the quantum *in silico* simulation with the 6-311+G(d,p) basis set. This functional belongs to one of the most accurate general-purpose GGAs, reaching, for example, for the G97/2 set of the heat of formations, a mean absolute deviation of only 3.8 kcal mol^−1^. The performance for noncovalently bound systems including many pure van der Waals complexes is excellent, reaching the average CCSD(T) accuracy (Grimme, 2006). Moreover, it improves the accuracy of describing the atom’s electronic structure and consequently, the molecular interactions and (relative) binding affinities, which are pivotal to macromolecular systems in the context of drug lead discovery and design (De Sousa et al., 2017; Bezerra et al., 2020; Vianna et al., 2021; Santos et al., 2022).

The effect of the residues surrounding formed by neighboring atoms (amino acids and water molecules) was included in our calculations using the conductor-like polarizable continuum model (CPCM) (Barone and Cossi, 1998; Cossi et al., 2003) through the dielectric constant $\varepsilon_{40}$ , which represents well the influence of electrostatic environment surrounding the residue-residue complex (Vicatos et al., 2009; Tavares et al., 2018; Campos et al., 2020; Morais et al., 2020).

### 2.2 Molecular fractionation with conjugate caps

As presented above, we fragmented the proteins into amino acids following the MFCC scheme, as in reference (Rodrigues et al., 2013). In the framework of this approach, for each amino acid of interest of the NSP12 at position $R_{i}$ , we mapped its distance to the residues in the proteins NSP7, NSP8_1_, and NSP8_2_ at position $R_{j}$ and chose those $R_{i}$ – $R_{j}$ pairs, that showed at least one atom inside a radius (r) equal to 8.0 Å. Thus, $R_{i}$ and $R_{j}$ were decomposed into individual fragments by cutting through the peptide bonds and a pair of conjugate caps was designed to saturate each fragment, aiming to preserve the local chemical environment and comply with the valence requirements. Here, the caps are formed by the neighbor residue covalently bound to the amine ( $C_{i}$ and $C_{j}$ ) and carboxyl ( $C_{i*}$ and $C_{j*}$ ) groups of residues $R_{i}$ and $R_{j}$ , respectively, along the protein chain. This provides a better description of its electronic environment. Hydrogen atoms are added to the molecular caps to avoid dangling bonds (He et al., 2014). Finally, the interaction energy (IE) of each residue-residue pair, IE( $R_{i}$ – $R_{j}$ ), was calculated as follows in Equation 1:

$$IE\left(R_{i}-R_{j}\right)=E\left(\Delta_{ij}\right)-E\left(\Delta_{i}\delta_{j}\right)-E\left(\delta_{i}\Delta_{j}\right)+E\left(\delta_{ij}\right),$$

where $\Delta_{m}$ = $C_{m}R_{m}C_{m*}$ and $\delta_{m}$ = $C_{m}C_{m*}$ ( m=i,j ). The term E( $\Delta_{ij}$ ) corresponds to the total energy of the fragment comprised of both capped residues. The second and the third terms of the equation, E( $\Delta_{i}\delta_{j}$ ) and E( $\delta_{i}\Delta_{j}$ ), give the total energy of the system formed by the capped residue $R_{i}$ and $R_{j}$ and the hydrogenated caps of $R_{j}$ and $R_{i}$ , respectively. E( $\delta_{ij}$ ) is the total energy of the system formed only by the caps. Additionally, to achieve the structural stability of the complex promoted by interactions with the extended hydration network, all water molecules forming hydrogen bonds with a particular residue or cap were included for completeness in the fragments. The descriptions of the interaction types were obtained through the Discovery Studio visualizer (Biovia Dassault Systèmes, 2021) and visual inspection.

## 3 Results and discussion

**FIGURE 2:**
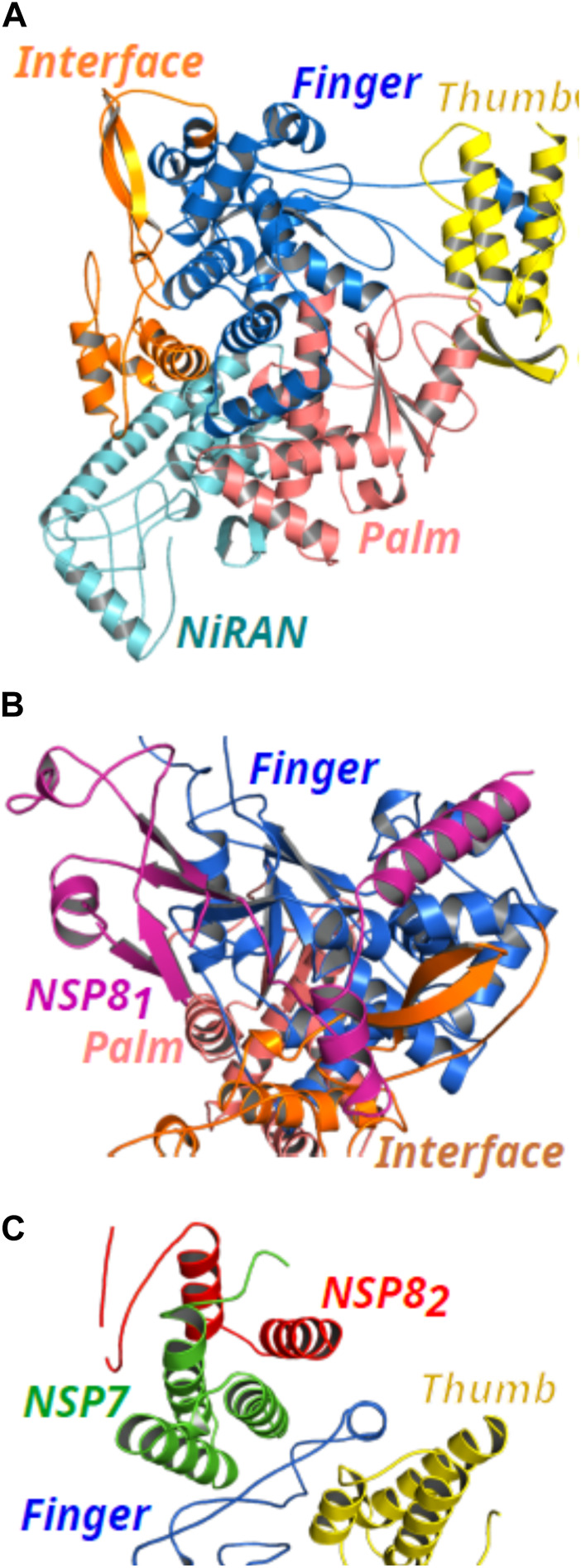
(Color online) Representation of the NSP12 protein and its binding site for NSP7 and NSP8 proteins (PDB ID: 6M71). **(A)** NSP12 domains are formed by NiRAN (cyan color), Interface (orange color), and the three subdomains of the C-terminal RdRp composed by Finger (marine-blue color), Palm (light-pink color), and Thumb (yellow color). **(B)** Cartoon representation of the NSP81 (magenta color) binding site in Finger and Interface subdomains. **(C)** Cartoon representation of the binding site of the NSP7 (green color) and NSP82 (red color) proteins in NSP12 Thumb and Finger subdomains.

In this work, quantum mechanical calculations were employed to describe residue-residue interactions and highlight the hotspots on the protein’s surface. This is a valuable strategy in drug design because it allows the identification of druggable sites. For the completeness of the analysis, not only residues located at the interface but all residues within 8.0 Å from the interface of the proteins were analyzed (Silva et al., 2020; Lima Neto et al., 2022). A schematic representation of the proteins is shown in Figure 2. The structure of the NSP12 (Figure 2A) is formed by two domains connected by an Interface domain (residues 250-365; orange): an N-terminal nidovirus RdRp-associated nucleotidyltransferase (NiRAN) domain (residues D60-R249; cyan), and a C-terminal right-handed RdRp domain (residues 366-920). The C-terminal RdRp is also divided into three subdomains: Finger (residues 366-580 and 620-678; marine-blue), Palm (residues 581-619 and 679-814; light-pink), and Thumb (residues 815-932; yellow), which is a conserved architecture in all viral RdRps (Padhi et al., 2021; Tian et al., 2021).

In Figures 2B, C, we present the binding site for NSP8_1_, NSP7, and NSP8_2_, respectively. As one can see, NSP8_1_ clamps the top region of the Finger subdomain (marine-blue color) and also forms additional interactions with the Interface domain (orange color). On the other hand, the NSP7-NSP8_2_ dimer binds above the Thumb subdomain (yellow color) of NSP12 and sandwiches the Finger loops, possibly to stabilize its conformation with NSP7 mediating most of the interactions in the dimer with NSP12, whereas, only a small region of NSP8_2_ looks to be in close contact with NSP12 residues.

Evaluating our results, we observed that in the supercomplex NSP12-NSP7-NSP8 solved in the *apo* form (PDB ID: 6M71), a total of 48 residues of NSP12, and 44 residues belonging to NSP7 were considered in the analysis of the PPIs in the dimer NSP12-NSP7. Furthermore, 122 residues of NSP12 and 88 residues of NSP8_1_ were analyzed in the dimer NSP12-NSP8_1_, resulting in 313 residue-residue interaction pairs with the energy calculated for the first dimer (NSP12-NSP7) and 866 for the second one (NSP12-NSP8_1_). The last PPIs studied in this PDB structure were in the NSP12-NSP8_2_ dimer, where NSP8_2_ contributed with 9 residues and NSP12 with 16, totalizing 38 interaction pairs. In the super complex NSP12-NSP7-NSP8 solved with the template-primer RNA and redeliver (PDB ID: 7BV2), 43 (87) residues of NSP7 (NSP8_1_), and 51 (126) residues of NSP12 (NSP8_1_) were considered in the analysis of the PPIs in NSP12-NSP7 (NSP12-NSP8_1_), resulting in 326 (849) residue-residue interaction pairs with energy calculated.

To facilitate the identification when we are referring to the analysis of the PPIs in the proteins of the PDB 6M71 or the 7BV2 crystals, we included the superscript APO and RNA in protein/residue names, respectively. The total interaction energy (TIE) of the dimers was calculated as the sum of pairwise interactions in each complex: NSP12-NSP7^APO^ ( −90.24 kcal mol^−1^), NSP12-NSP7^RNA^ ( −85.28 kcal mol^−1^), NSP12-NSP8_1_^APO^ ( −320.93 kcal mol^−1^), NSP12-NSP8_1_^RNA^ ( −327.58 kcal mol^−1^), and NSP12-NSP8_2_^APO^ ( −19.44 kcal mol^-1^).

Unfortunately, as we know, there is no experimental data to compare with these energy outcomes. However, (Sarma et al., 2022), calculated the binding free energy (BFE) of the two dimers, NSP12-NSP7 and NSP12-NSP8 (PDB ID: 6M71), through molecular dynamics simulation and the MM/PBSA scheme, showing that the second dimer has a stronger binding energy than NSP12-NSP7, as we observed for both structures here studied. (Wilamowski et al., 2021) observed that the NSP12-NSP7-NSP8 super complex is more stable after the binding of the RNA molecule, but they suggested that NSP7 and NSP8 are static components, helping to close the complex once RNA is bound, guiding the RNA upon exit, and stabilizing contacts with other proteins in the replication/transcription process. Our energetic results were quite close between the *apo* form, and that bound to the RNA+remdesivir. Hence, further studies are necessary to understand the stability of the supercomplex in the presence/absence of RNA, as well as with RNA plus the inhibitor remdesivir.

It is worth mentioning that the central idea of this work is not to supply the complete binding mechanism but to describe the intermolecular interaction energies between NSP12-NSP7/NSP8, identifying the most important interactions present in the crystallographic structures. Thus, our computational results correspond to a view of the static molecular momentum of these proteins. It is important to mention that beyond the interaction energy (enthalpic effect) here obtained, protein-protein binding is also governed by other factors, including (de)hydration, hydrophobic effects, and entropic contributions, and the interaction between residues of the same protein (intramolecular) that could also help in the stabilization of a certain structural conformation, not considered in this work. These can induce changes in the solvent interaction interface and the formation or breaking of intermolecular interactions.

Taking into account the dynamic nature of the biomolecules, a classical molecular dynamics (MD) approach, with adequate phase space sampling, might provide a more realistic dynamic structure. However, unfortunately, it gives poor accuracy in describing the interaction energies depicted in this work, as compared to a quantum chemistry approach (Ryde and Söderhjelm, 2016). Therefore, we have used molecular quantum chemistry calculations based on the DFT scheme, which is a route to investigate accurately large biological systems with affordable computational cost. It has been successfully employed previously to describe ligand-protein interactions at the quantum level (for a review see the recent book (Albuquerque et al., 2021), and the references therein).

One of the most significant properties of the PPI interface is that the energy is not uniformly distributed, and the hotspot residues have the greatest impact on binding energy in the protein complex (Bogan and Thorn, 1998). To evaluate the hotspot residues across the PPI interfaces in the dimers NSP12-NSP7 and NSP12-NSP8, we calculated all the residue-residue interaction energy (IE) within a *r* equal to 8.0 Å and presented the most energetically significant ones below. Here, the results are presented and discussed following the order NSP12-NSP7, NSP12-NSP8_1_, and NSP12-NSP8_2_. When the dimer is present in both crystal structures, they are also compared in the same subsection.

### 3.1 Analysis of the NSP12–NSP7 dimer interactions

Table 1 depicts the 13 interaction pairs showing an energy value stronger than 2.0 or −2.0 kcal mol^−1^ at least in one of the two complexes studied. From these, 8 interaction pairs were within the energy cutoff in the complex NSP12-NSP7^APO^, with the strongest interaction energies (IEs) found in the pairs A443-N37^APO^, and F415-C8^APO^, while the weakest IEs were found in the pairs F429-S4^APO^, and E445-V33^APO^. In the dimer NSP12-NSP7^RNA^, the strongest IEs were observed in the pairs E431-K2^RNA^, and F440-L40^RNA^, while the weakest interaction energies were observed in the pairs F442-L41^RNA^, and Q444-W29^RNA^. The residue-residue pairs showing the strongest interaction energies have the IE highlighted in bold red, whereas those showing the weakest IEs were in bold. Besides, the IE of the pair A443-N37^RNA^ is in bold blue because it is the only residue-residue pair presenting positive (repulsion) interaction energy among the 13 selected.

**TABLE 1:** Energy values (in kcal mol^−1^) of the most energetically relevant residue-residue interaction pairs for the dimer NSP12-NSP7.

NSP12–NSP7 residues	Energy (kcal mol^−1^)	
	NSP12-NSP7^APO^	NSP12-NSP7^RNA^
K411–Q18	−2.57	−1.71
K411–E23	−2.57	−1.23
F415–C8	**−3.78**	−3.10
Y420–S4	−2.76	−1.62
F429–S4	−2.04	−1.91
E431–K2	−2.32	**−3.63**
F440–K7	−1.85	−2.15
F440–L40	−1.81	**−3.24**
F442–L40	−1.97	−2.15
F442–L41	−1.19	−2.07
A443–N37	**−4.28**	**2.46**
Q444–W29	−1.66	−2.08
D445–V33	−2.09	−1.39

The strongest attractive (repulsive) interaction energies are shown in bold red (bold blue).

**FIGURE 3:**
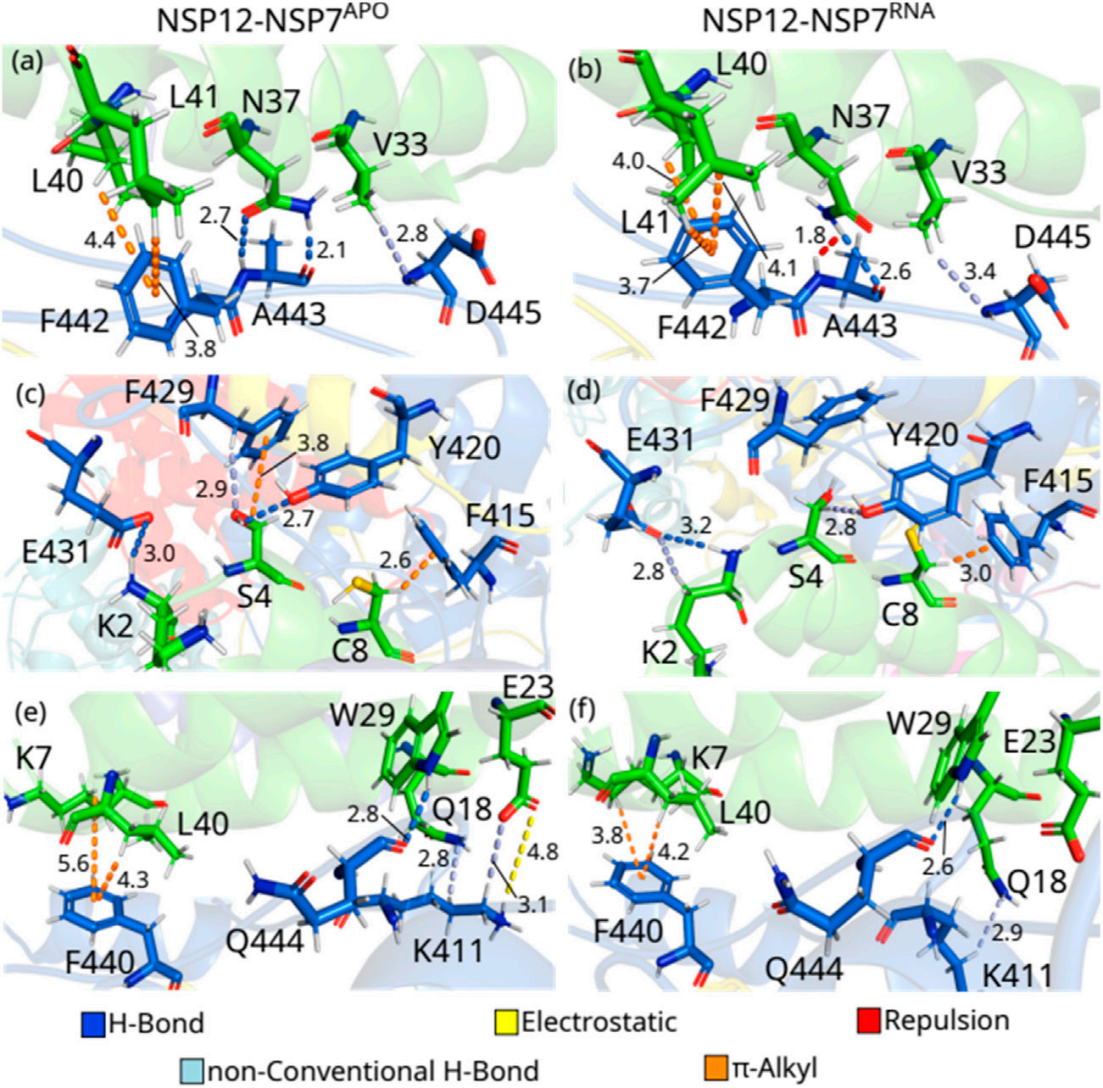
(Color online) Detailed spatial arrangement of the major NSP12-NSP7 interaction pairs with their intermolecular interaction in PDB ID 6M71 [left - **(A)**, **(C)** and **(E)**] and 7BV2 [right **(B)**, **(D)** and **(F)**]. Dashed lines in marine (light-blue) represent direct (non-conventional) hydrogen bonds, while orange (yellow) lines represent π-alkyl (electrostatic) interactions. Repulsion is represented by red lines.

As one can see, by comparing NSP12-NSP7^APO^ with NSP12-NSP7^RNA^, most of the IEs are quite close, indicating a similar interaction pattern, and that only small shifts occurred in the protein after the introduction of the RNA+remdesivir to the complex. In Figure 3, we depict the most energetically relevant interaction pairs of NSP12-NSP7^APO^ (left) and NSP12-NSP7^RNA^ (right). Three types of intermolecular interactions dominate the residue-residue pairs: hydrogen bonds (H-bonds), non-conventional H-bonds, and π -alkyl, albeit an electrostatic (ion-ion) interaction and a repulsion were also observed. We present these interactions below, followed by a comparison between the two complexes studied.

A443-N37^APO^ is the residue-residue pair showing the strongest interaction energy among those analyzed from both complexes. The polar atoms of the alanine main chain make two H-bonds with the two polar atoms of the asparagine’s side chain at 2.1 and 2.7 Å (Figure 3A). On the other hand, the pair A443-N37^RNA^ is the only one presenting a positive (repulsive) IE. Looking at Figure 3B, one can see that the side chain of N37 rotates (see Supplementary Figure S1A), so its group NH^2^ is close to the main chain NH of N443 (1.8 Å) forming a repulsion between the residues, whereas an H-bond is also formed (2.6 Å). In Supplementary Figure S2, we present the electrostatic potential map showing the region in blue depicting the repulsion between the NH groups (blue), as well as between the oxygen atoms (red) of the residues. According to (Biswal et al., 2021), the mutation of N37 to valine (N37V) greatly hampers RdRp activity of SARS-CoV-2 NSP12-NSP7-NSP8 since it disrupts the hydrogen bond formed with A443, but the stability of NSP7–NSP8 is maintained. (Subissi et al., 2014) performed a mutation in N37 (to alanine) of SARS-CoV, observing that it impairs the RdRp activity of the protein, which reinforces the conservation of the binding site of NSP12-NSP7 among different CoVs. Noteworthy (Sarma et al., 2022), evaluated the binding of these proteins through molecular dynamics simulation together with MM/PBSA analysis, finding that N37 plays an important role in the formation of NSP12-NSP7 complex in SARS-CoV-2 through the formation of H-bonds, although its role in SARS-CoV was not completely elucidated.

D445-V33^APO^ presents one of the weakest IE within our energy cutoff, and the interaction energy of D445-V33^RNA^ is still weaker. In both cases, the residues are making a non-conventional H-bond between the main chain of D445 and the side chain of V33, but at different distances, with the first at 2.8 Å and the second at 3.4 Å (Figures 3A, B). In the pairs, F442-L40 and F442-L41 occur the opposite of what has been observed so far, with the NSP12-NSP7^APO^ pair showing a weaker IE than the NSP12-NSP7^RNA^ one. F442-L40^APO^ forms a π -alkyl interaction with a distance of 4.4 Å, while the same interaction is found in F442-L40^RNA^, but at a shorter distance (4.0 Å). Similarly, the pair F442-L41^APO^ also makes a π -alkyl interaction (3.8 Å), and there is a reorganization in the side chain of F442, as well as in L41 in the crystal with RNA (see Supplementary Figure S1A) that allows the formation of a second π -alkyl interaction (3.7 and 4.1 Å). As we know, the relevance of these residues was not previously reported.

F415-C8^APO^ shows one of the strongest IE, and it is quite close to the interaction energy found in the pair F415-C8^RNA^. In both cases, these residues are making a π -alkyl interaction at the distance of 2.6 Å (Figure 3C) and 3.0 Å (Figure 3D), respectively, besides some other hydrophobic contacts. It was previously shown that the mutation of the residue C8 to glycine (C8G) leads to a severe reduction of RdRP efficiency since it interacts with residues at the interface of NSP8 and NSP12 (Biswal et al., 2021), as well as (Sarma et al., 2022) observed the relevance of this residue not only for SARS-CoV-2 but also to SARS-CoV.

S4^APO^ interacts with Y420^APO^ and F429^APO^ and presents IE within our energy criteria. As one can see in Figure 3C, S4^APO^ is forming an H-bond with Y420^APO^ (Y420-S4^APO^: 2.7 Å), while a π -alkyl (3.8 Å) interaction and a non-conventional H-bond (2.9 Å) are made with F429^APO^. On the other hand, there is a rotation in the side chain of S4^RNA^ (see Supplementary Figure S1B) that impairs the formation of the same intermolecular interactions. Thus, Y420-S4^RNA^ is making a non-conventional H-bond (2.8 Å), while only small hydrophobic contacts were observed in the pair F429-S4^RNA^. Despite the residue serine 4 being part of the interface interacting with NSP8 and NSP12, we do not find any other study showing the role of this residue in the activity of SARS-CoV-2 NSP12-NSP7-NSP8 supercomplex, although the Ref. (Sarma et al., 2022) also showed Y420 and F429 as important residues to the interface.

E431-K2^RNA^ is the strongest interaction energy among the residue-residue pairs studied in the NSP12-NSP7^RNA^ dimer, and the third by comparing all the pairs in this section, whereas E431-K2^APO^ shows the fifth-strongest IE of NSP12-NSP7^APO^. As one can see in Supplementary Figure S1B, these residues are rearranged in E431-K2^RNA^, which leads to the formation of not only one H-bond (3.2 Å; Figure 3D), as in E431-K2^APO^ (3.0 Å; Figure 3C), but also a non-conventional H-bond (2.8 Å; Figure 3D), increasing the interaction energy of the pair. K411^APO^ interacts with Q18^APO^ and E23^APO^, making a non-conventional H-bond with both of them at a distance of 2.8 Å and 3.1 Å (Figure 3E), respectively, while it also forms an electrostatic interaction with E23^APO^ (4.8 Å) and some hydrophobic interactions with Q18^APO^. However, the position of the side chain of K411^RNA^ shifts and moves away from E23^RNA^ in NSP12-NSP7^RNA^ and no direct interaction was observed, while the non-conventional H-bond with Q18^RNA^ is maintained (2.9 Å; Figure 3F).

**FIGURE 4:**
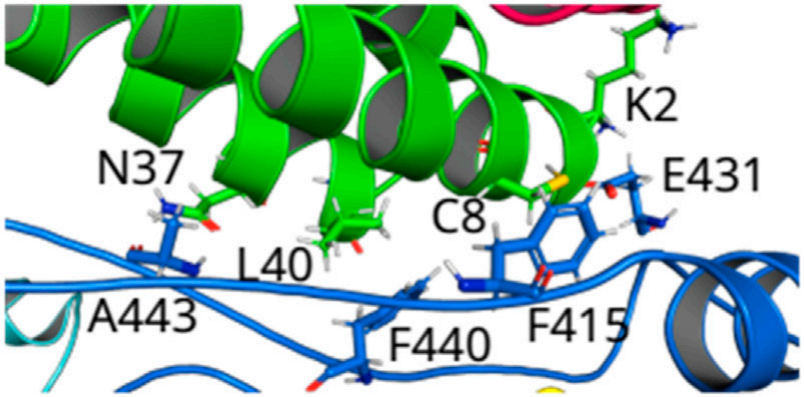
(Color online) Overview of the loops connecting Finger-Thumb regions from NSP12 to the helices of NSP7. Hotspot regions in NSP12--NSP7 formed by the amino acids that make hydrogen bonds (E431--K2 and A443--N37) and -alkyl interactions (F415--C8 and F440—L40).

Q444-W29 are forming H-bonds at 2.8 and 2.6 Å in NSP12-NSP7^APO^ and NSP12-NSP7^RNA^, respectively (Figures 3E, F). F440 interacts with K7 and L40 through a π -alkyl interaction in both complexes here analyzed (Figures 3E, F). Interestingly, in both cases, the IEs observed in these pairs are stronger in NSP12-NSP7^RNA^ than NSP12-NSP7^APO^ with interaction distances shorter in the first one. The key role of F440, K7, and L40 for the binding energy of the complex was shown by (Sarma et al., 2022), while the residue K7 of SARS-CoV (NSP7) is found to be essential for RdRp activity (Subissi et al., 2014). Thus, we observed that the two loops that connect the Finger-Thumb regions of NSP12 and the top of the helices that form NSP7 are very relevant to the interaction between these to protein (see Figure 4), as well as break the H-bonds in the pairs E431-K2 and A443-N37, and the π -alkyl interactions in the pairs F415-C8 and F440-L40 could be important to impair the RdRp activity of the complex.

### 3.2 Analysis of the NSP12–NSP8_1_ and NSP12–NSP8_2_ interactions

By analyzing the individual interaction energy of the residue-residue pairs in the dimer NSP12-NSP8^1^, we observed that more than 50 pairs showed IE over the energy criteria used in the last subsection, i.e., stronger than 2.0 or −2.0 kcal mol^-1^ at least in one of the two complexes studied (see Supplementary Table S1). Therefore, we increased the IE cutoff to values stronger than 4.0 or −4.0 kcal mol^-1^ to present only the most relevant interactions to the complex. Hence, 17 residue-residue pairs were selected and are shown in Table 2. From these, 14 interaction pairs were within the energy cutoff in the complex NSP12-NSP8_1_^APO^, with the strongest interaction energies (IEs) found in the pairs K332-D99^APO^, and R331-D112^APO^, while the weakest IEs were found in the pairs S384-K97^APO^, and L514-K79^APO^. In the dimer NSP12-NSP8_1_^APO^, the strongest IEs were observed in the pairs K332-D99^RNA^, and L389-V130^RNA^, while the weakest interaction energies were observed in the pairs R331-D112^RNA^, and D517-K79^RNA^. The residue-residue pairs showing the strongest interaction energies have the IE highlighted in bold red, whereas those showing the weakest IEs were in bold. Besides, the pair S384-M94^APO^ is in bold blue because it is the only residue-residue pair presenting positive (repulsion) interaction energy among the 17 selected.

**TABLE 2:** Energy values (in kcal mol^−1^) of the most important residue-residue interaction pairs for the dimer NSP12-NSP8.

NSP12–NSP8_1_	Energy (kcal mol^−1^)	
Residues	NSP12-NSP8_1_^APO^	NSP12-NSP8_1_^RNA^
L514–K79	−3.43	−4.71
D517–K79	−4.42	−4.38
D523–R80	−1.22	−5.36
S384–M94	**5.33**	−2.48
S384–K97	−3.51	−5.71
G385–K97	−4.12	−2.60
K332–D99	**−14.74**	**−11.63**
K332–N104	−6.45	−2.22
H355–R111	−5.77	−0.16
R331–D112	**−8.16**	−4.66
R331–G113	−4.80	−0.65
R331–C114	−4.72	0.49
L329–V115	−4.40	−4.76
N386–K127	−0.78	−5.45
L387–L128	−5.62	−6.45
L388–M129	−1.66	−5.71
L389–V130	−5.30	**−6.53**

The strongest attractive (repulsive) interaction energies are shown in bold red (bold blue).

**FIGURE 5:**
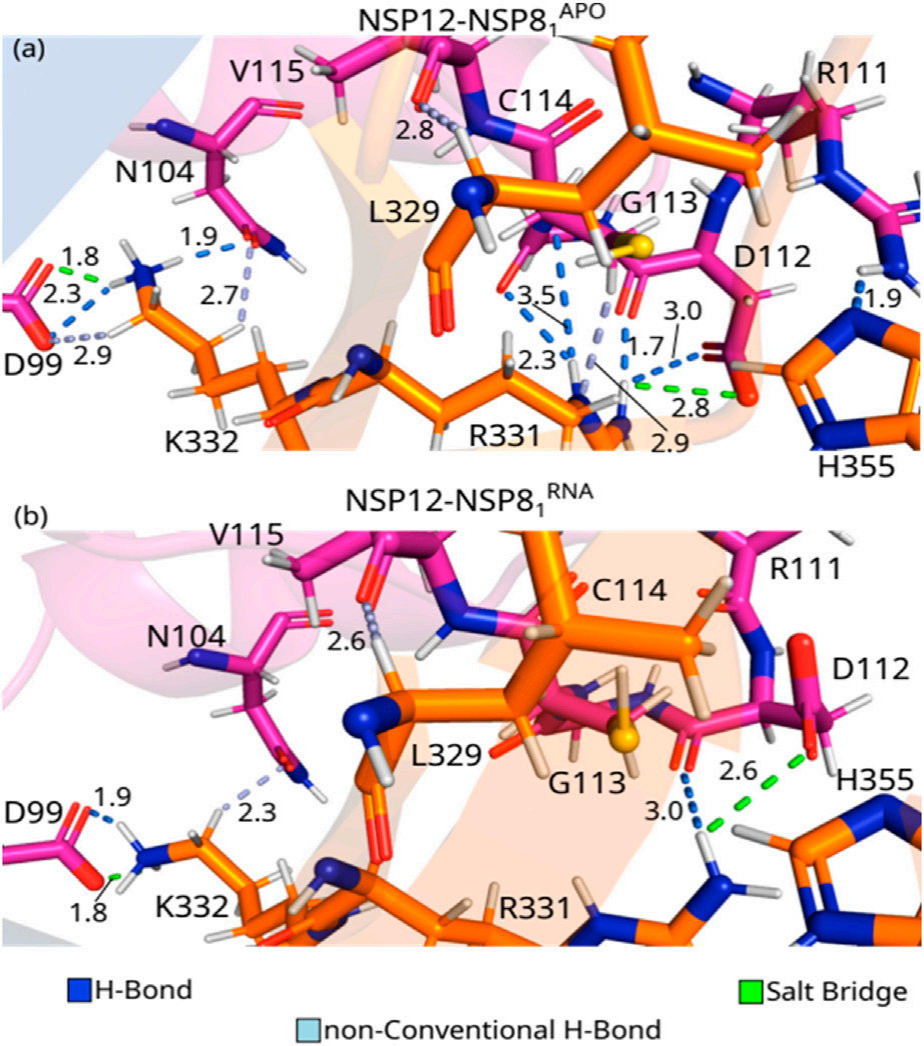
(Color online) Detailed spatial organization of the NSP12(Interface)-NSP81 most relevant interaction pairs with their intermolecular interaction. Dashed lines in marine (light blue) represent direct (non-conventional) hydrogen bonds and salt bridge is represented in green lines. **(A)** Depicts PDB ID: 6M71, and **(B)** the PDB ID: 7BV2.

As one can see, contrary to what was previously observed in the NSP12-NSP7 dimer, the difference between most of the IEs (11) in NSP12-NSP8_1_^APO^ and NSP12-NSP8_1_^RNA^ are > 2.0 kcal mol^−1^, indicating that changes might have occurred in the interaction’s pattern after the introduction of the RNA+remdesivir to the complex. Besides, two regions of NSP12 (Interface and Finger) interact with NSP8_1_ protein with IEs within our energy cutoff value. In Figure 5, we show the interaction pairs between the NSP12 (Interface) and NSP8_1_: K332-D99, K332-N104, H355-R111, R331-D112, R331-G113, R331-C114, and L329-V115.

K332-D99^APO^ is the residue-residue pair showing the strongest IE among the three complexes studied in this paper. As one can see in Figure 5A, these residues are making a salt bridge (1.8 Å), as well as an H-bond (2.3 Å), and a non-conventional H-bond (2.9 Å). On the other hand, the pair K332-D99^RNA^ shows the second-strongest IE, forming a salt bridge (1.8 Å), and an H-bond (1.9 Å), see Figure 5B. K332^APO^ also interacts with N104^APO^ through an H-bond (1.9 Å) and a non-conventional H-bond (2.7 Å; Figure 5A), whereas K332-RN104^RNA^ only forms non-conventional H-bond (2.3 Å; Figure 5B). As shown in Supplementary Figure S1C, the shift in the side-chain of the lysine residue hindered the formation of the non-conventional H-bond in the K332-D99^RNA^ pair, as well as the H-bond with N104^RNA^, which decreased its interaction energy, when compared to the crystal in the *apo* form. The per-residue energy contribution of N104, D99, and K332 was previously observed by Sarma et al. (2022) for SARS-CoV and SARS-CoV-2. The mutation D99A was observed to generate crippled *in vivo* phenotypes of SARS-CoV, with reduced plaque size and lower progeny titers (Subissi et al., 2014), that is because it disrupts the electrostatic interaction with K332 (Kirchdoerfer and Ward, 2019). Furthermore, no experimental study has shown the relevance of N104 yet.

R331-D112^APO^ presents one of the strongest IEs, and it is almost double the value of R331-D112^RNA^. As one can see in Figure 5A, the side-chains of the pair R331-D112^APO^ are making two H-bonds (1.7 and 3.0 Å), and a salt bridge (2.8 Å), whereas the residues R331-D112^RNA^ are forming a salt bridge (2.6 Å), and an H-bond (3.0 Å; Figure 5B). R331^APO^ is also forming an H-bond with G113 ^APO^ (2.3 Å) and C114^APO^ (3.5 Å), a non-conventional H-bond (2.9 Å) and hydrophobic interactions with the residue C114^APO^, see Figure 5A. Similar to what we observed in residue K332, the side-chain of R331 shifts, decreasing the number of interactions formed between the residues in the dimer with the presence of RNA (Supplementary Figure S1C). This shift in the side-chain of R331^RNA^ makes it impossible to form direct contact with G113^RNA^ and C114^RNA^. Despite the strong interaction energy, the relevance of these residues was not tested experimentally, as the authors know, but (Sarma et al., 2022) obtained a high value of energy contribution for both residues in SARS-CoV-2.

L329-V115^APO^ and H355-R111^APO^ are among the pairs showing the strongest interaction energies. The IE between the residues L329-V115 is quite similar in both crystal structures, which corresponds to the formation of a non-conventional H-bond at the distance of 2.8 Å and 2.6 Å in NSP12-NSP8^APO^ and NSP12-NSP8_1_^RNA^, respectively. The residues in the pair H355-R111^APO^ are interacting by an H-bond (1.9 Å; Figure 5A). On the other hand, in the NSP12-NSP8_1_^RNA^ crystal, the shift in the side-chain position of R111 (Supplementary Figure S1C) makes H355-R111^RNA^ so far away that they no longer interact.

**FIGURE 6:**
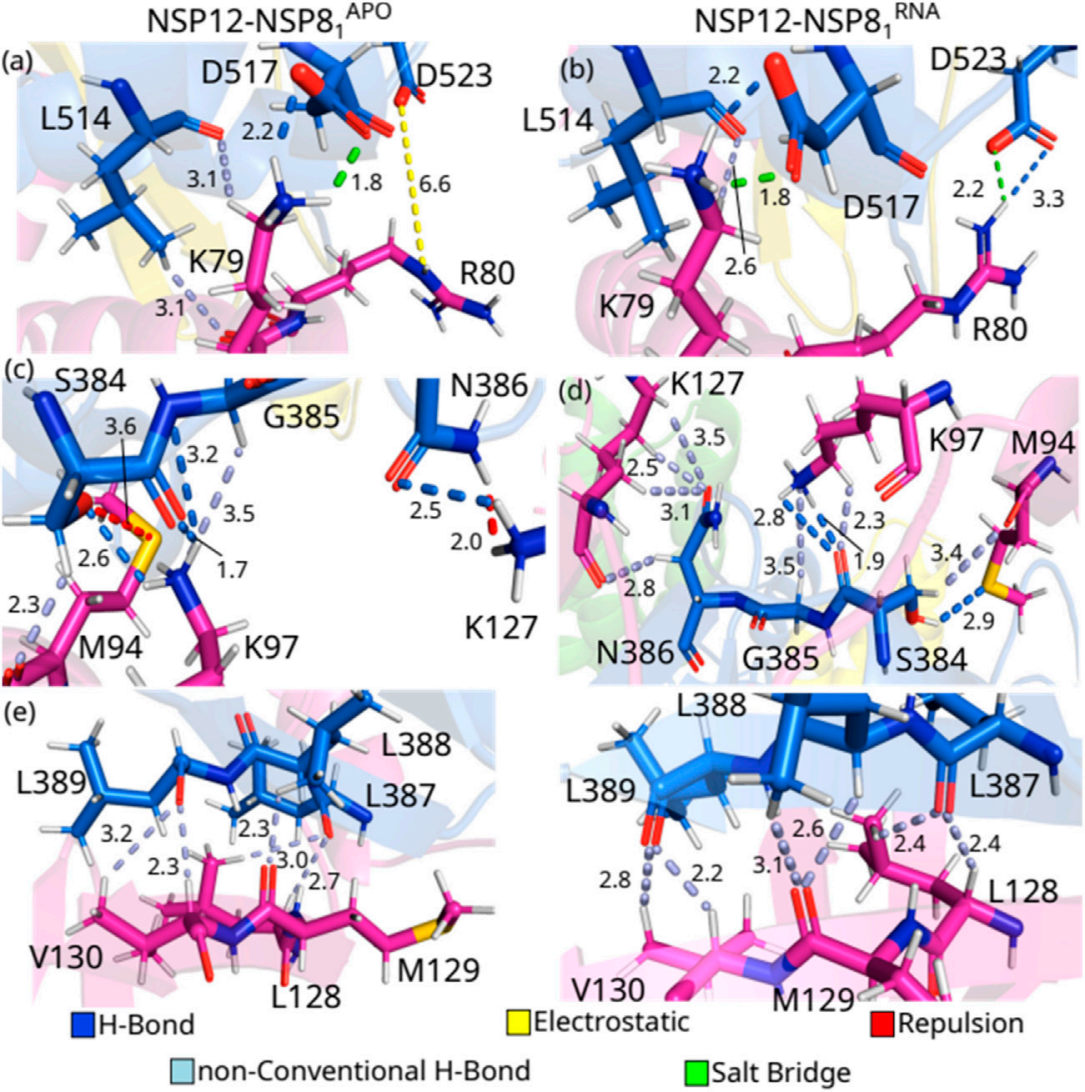
(Color online) Detailed spatial arrangement of the major NSP12(Finger)-NSP81 interaction pairs with their intermolecular interaction in PDB ID 6M71 [left - **(A)**, **(C)** and **(E)**] and 7BV2 [right **(B)**, **(D)** and **(F)**]. Dashed lines in marine (light-blue) represent direct (non-conventional) hydrogen bonds, while green (yellow) lines represent salt bridge (electrostatic) interactions. Repulsion is represented by red lines.

When the residues in the Interface region of NSP12 were taken into account, the calculated IEs were stronger for the crystal NSP12-NSP8_1_^APO^ compared to the NSP12-NSP8_1_^RNA^, while the opposite behavior can be observed in the interaction between the Finger region of the NSP12 protein and the NSP8_1_ protein. In Figure 6, we depict the most relevant interaction pairs between the NSP12 (Finger) and NSP8_1_: L514-K79, D517-K79, D523-R80, S384-M94, S384-K97, G385-K97, N386-K127, L387-L128, L388-M129, L389-V130.

One can see from Figure 6A that the residues L514-K79^APO^ form two non-conventional H-bonds at a distance of 3.1 Å each, and also hydrophobic interactions, while in the crystal with the RNA sequence, the hydrophobic interaction is maintained, but only one non-conventional hydrogen bond is observed, at a distance of 2.6 Å (Figure 6B). Residues D517 and K79 are making a salt bridge (1.8 Å) and an H-bond (2.2 Å) in both crystal structures. On the other hand, when we evaluated the interactions between the residues D523 and R80, we observed that the side-chain of D523-R80^APO^ is involved in electrostatic contacts, whereas a salt bridge (2.2 Å) and an H-bond (3.3 Å) are present in the crystal NSP12-NSP8_1_^RNA^.

S384 shows strong IEs when interacting with M94 and K97. Interestingly, S384-M94^APO^ presents the strongest positive (repulsion) energy, and it occurs even with the formation of non-conventional H-bonds (2.3 Å). Looking at Figure 6C, one can observe that the oxygen atom from the serine side-chain and the sulfur atom from the methionine residue are very close to each other (3.6 Å), while in the NSP12-NSP8_1_^RNA^ crystal (Figure 6D), the hydroxyl group of the serine residue is making an H-bond (2.9 Å) with M94 besides the non-conventional H-bond (3.4 Å). By interacting with K97^APO^, S384 makes two H-bonds (1.7 and 2.6 Å) with its side-chain NH^+^_3_ group, whereas it forms two H-bonds (1.9 and 2.8 Å) and a non-conventional H-bond (3.5 Å) with K97^RNA^ The residue K97 of NSP8_1_ also interacts strongly with G385^APO^, creating an H-bond (3.2 Å) and a non-conventional H-bond (3.5 Å). On the other hand, K97^RNA^ just makes a non-conventional H-bond (3.5 Å) with G385^RNA^. Moreover, N386-K127^APO^ are forming an H-bond (2.5 Å) and repulsion between their NH side-chain groups (2.0 Å), and N386–K127^RNA^ are making 4 non-conventional H-bonds (2.5, 2.8, 3.1, and 3.5 Å).

Finally, the region formed by 3 leucines (L387, L388, and L389) in NSP12 shows the strongest interaction energies among the evaluated pairs of NSP12 (Finger)-NSP8_1_. As one can see from Figures 6E, F, L387-L128 and L389-V130 are involved in two non-conventional H-bonds and hydrophobic interactions in both crystals, as well as L388-M129 form one non-conventional H-bond in NSP12-NSP8_1_^APO^ and two non-conventional H-bonds in NSP12-NSP8_1_^RNA^. Despite the energetic relevance of the residues here presented, we could not find them in other experimental or computational studies, except in the paper of (Sarma et al., 2022). Hence, future works could be performed to evaluate the role of these residues in the function of the complex.

**FIGURE 7:**
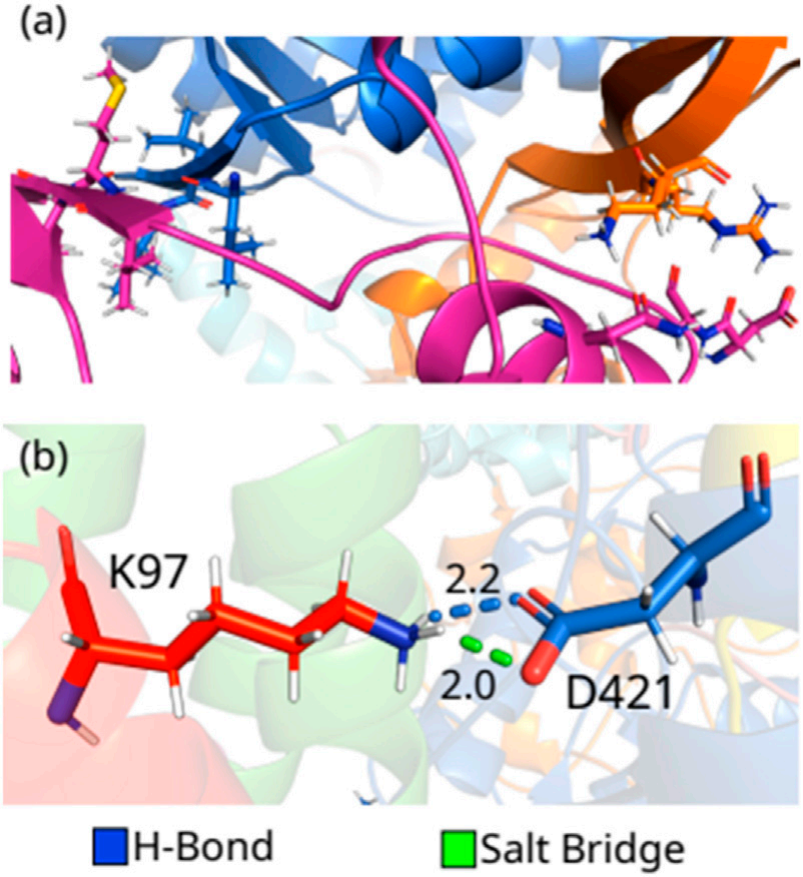
(color online) **(A)** Hotspot regions in NSP12-NSP81. **(B)** Detailed spatial organization of the NSP12-NSP82 most relevant interaction pair with the intermolecular interactions.

Thus, we observed that the region of the protein in which two positively charged residues (R331 and K332; Interface) are, forming strong interactions (salt bridge and H-bond) with D99, N104, and D112, could be a relevant target to break the interaction between NSP12 and NSP8_1_, as well as the region formed by the 3 leucine residues (L387, L388, and L389; Finger) that are related to the formation of several non-conventional H-bonds. These residues of NSP12 interact with a region of NSP8_1_ composed of the residues 99-130, mainly the residues in the helix and the β -strand (see Figure 7A).

In the end, we included the interaction between the proteins in the dimer NSP12-NSP8_2_ here because only one residue-residue pair showed interaction energy stronger than 2.0 or −2.0 kcal mol^-1^. D421-K97 ( −11.62 ) are making a salt bridge (2.0 Å) and an H-bond (2.2 Å), as in Figure 7B, contributing approximately 58% of the total interaction energy of the complex. It is important to mention that the dimer NSP12-NSP8_2_ is only present in the crystal with ID: 6M71, i.e., the NSP12-NSP8_2_^APO^. In Supplementary Tables S2, S3, we resume the interactions made by the energetically most relevant residue-residue pairs in NSP12-NSP7 and NSP12-NSP81, respectively.

## 4 Conclusion

The indiscriminate spread of the coronavirus poses a threat to human health around the world. NSP12 is the core component of the replication and transcription machinery of SARS-CoV-2, and the proteins NSP7 and NSP8 are essential for its function. This suggests that compounds that could disrupt the binding of NSP7 or NSP8 to the NSP12 complex might help to fight against the virus. However, there are few residues at this complex’s interface identified as probable hotspots. We used quantum biochemistry methods to investigate the interactions between the proteins in the dimers NSP12-NSP7, NSP12-NSP8_1_, and NSP12-NSP8_2_, in atomic detail to understand the process by which these proteins interact. We aimed to discover hotspots that could be used to neutralize viral infection.

According to the protein-protein interaction results, the total interaction energy follows the order: NSP12-NSP8_1_ > NSP12-NSP7 > NSP12–NSP8_2_. Evaluating the individual interaction energies (IEs) between the residues, 14 pairs of NSP12-NSP8_1_ presented the strongest IEs: L514-K79, D517-K79, S384-M94, S384-K97, G385-K97, K332-D99, K332-N104, H355-R111, R331-D112, R331-G113, R331-C114, L329-V115, L387-L128, and L389-V130. In the complex NSP12-NSP7, we find 8 residue-residue pairs with strongest IEs (K411-Q18, K411-E23, F415-C8, Y420-S4, F429-S4, E431-K2, A443-N37, and D445-V33), whereas only one pair (D421-K97) proved to be energetically relevant. Besides, we observed that hydrophobic interactions are key for the dimer NSP12-NSP7, while hydrogen bonds are the most relevant for NSP12-NSP8. Unfortunately, as far as we know, there is no experimental data to compare with our total interaction energy outcomes. On the other hand, we have found only two experimental papers in which the relevance of the residues is taken into account through their mutation for the SARS-1 (Subissi et al., 2014) and SARS-2 (Biswal et al., 2021) NSP12-NSP7-NSP8 complex. Thus, new studies are necessary to understand this complex better, and our analysis could help to present new residues that could be relevant to the complex.

Two major hotspots in NSP12 were found, formed by the residues F415, E431, F440, and A443, and R331, K332, L387, L388, and L389. Our data suggest that designing an inhibitor for impairing the contacts between the residues in the interface NSP12-NSP8_1_ could be more interesting than NSP12-NSP8_2_, albeit NSP12-NSP7 could also be an option, mainly in the regions shown in Figures 4, 7A. Finally, the introduction of RNA+remdesivir in the complex almost does not alter the IE of the residue-residue pairs of NSP12-NSP7, while changes are more evident in NSP12-NSP8_1_. These results provide valuable information for the discovery of antiviral therapeutics that inhibit these protein-protein interactions in human pathogenic CoVs.

## Data Availability

The original contributions presented in the study are included in the article/Supplementary Material, further inquiries can be directed to the corresponding authors.
