# Supplementary material for: Investigation of protein-protein interactions and hotspot region on the NSP7-NSP8 binding site in NSP12 of SARS-CoV-2
Source: Front Mol Biosci. 2024 Jan 18;10:1325588. doi: 10.3389/fmolb.2023.1325588 (PMC10830813; doi:10.3389/fmolb.2023.1325588)
Supplement: Supplementary file 1 [file DataSheet1.docx]

Investigation of protein-protein interactions and hotspot region on the NSP7-NSP8 binding site in NSP12 of SARS-CoV-2

Supplementary Material

(a)


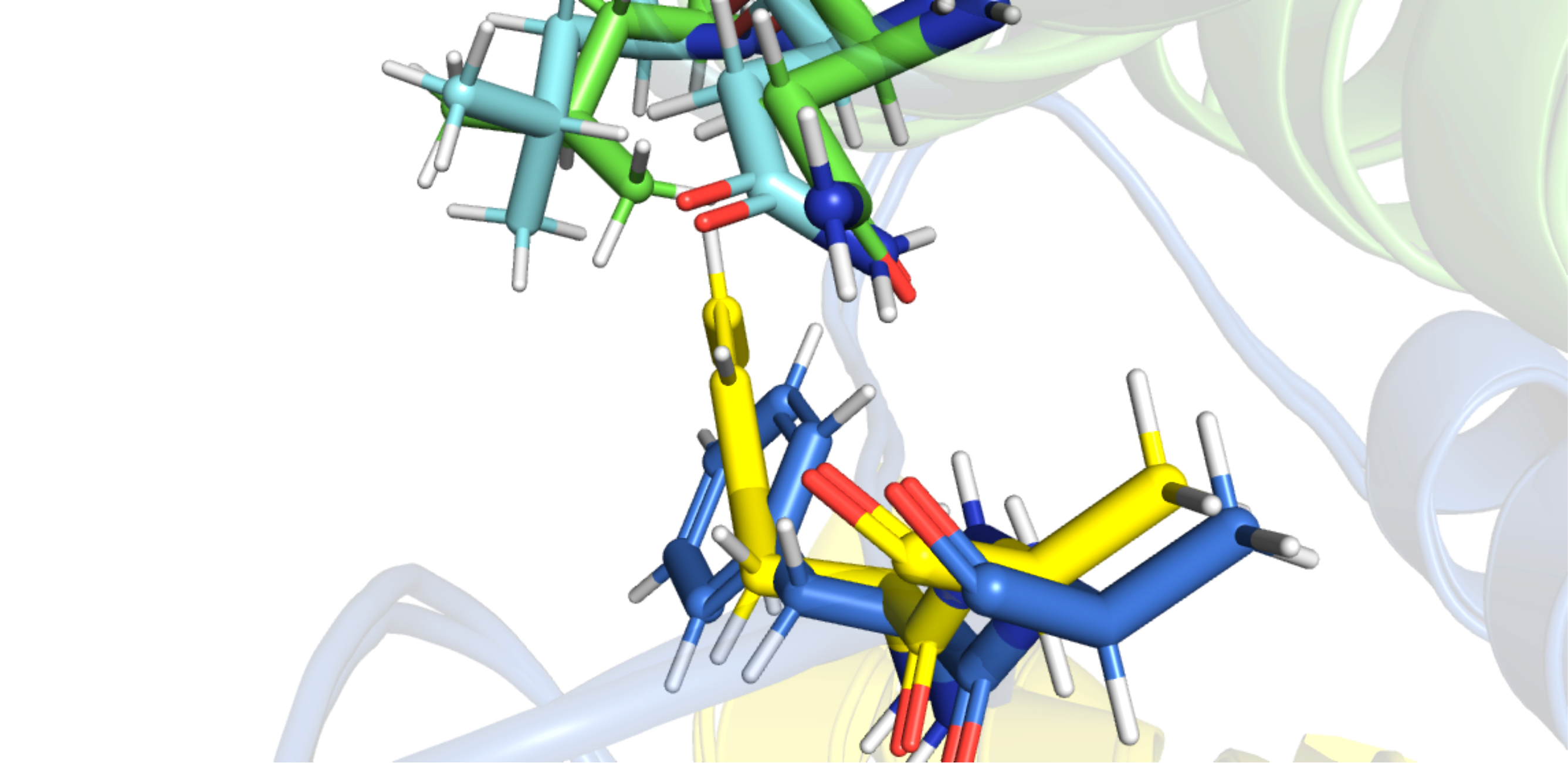

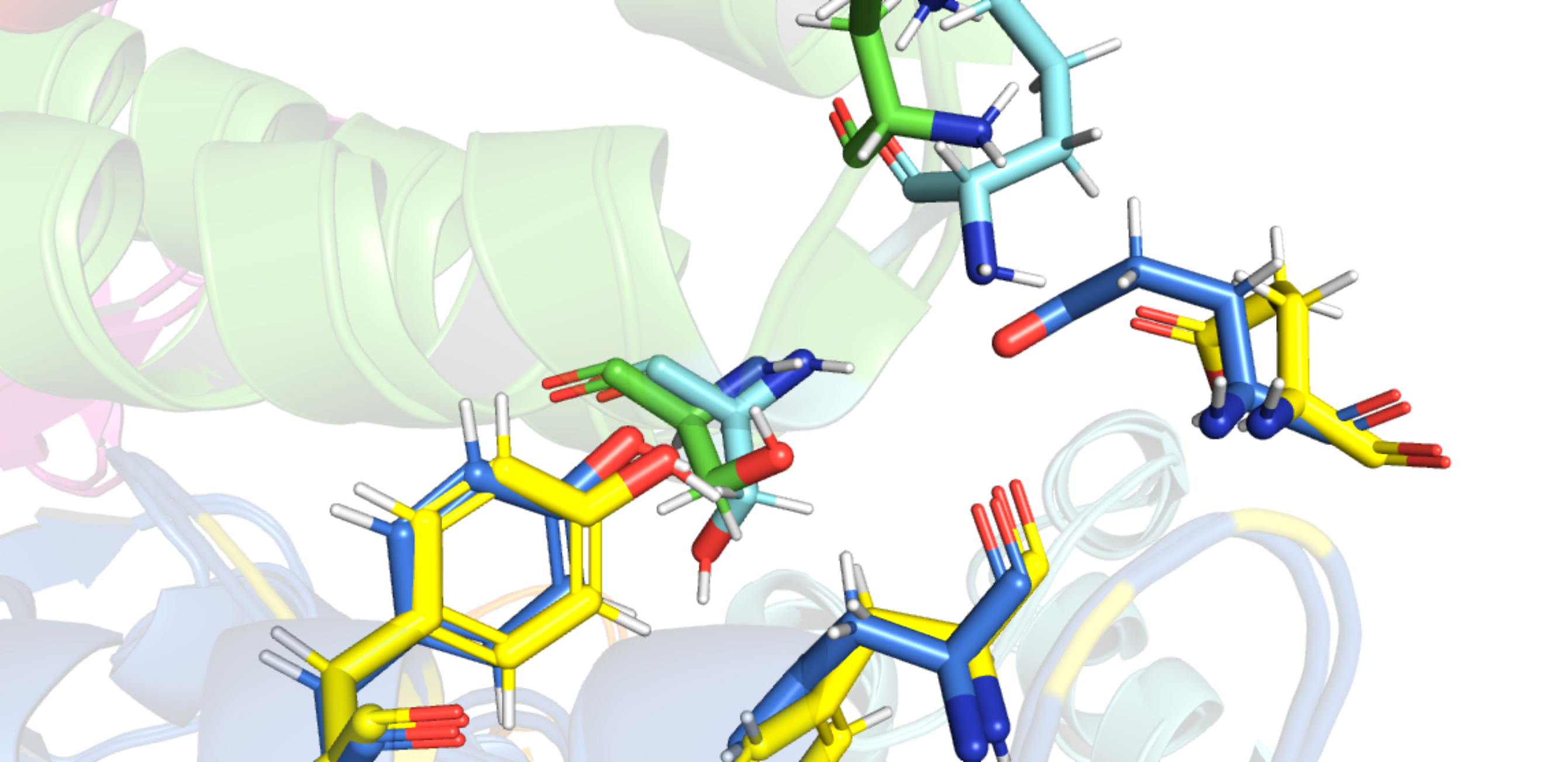


L41

N37

NSP7APO NSP7RNA NSP12APO NSP12RNA NSP8_1_APO NSP8_1_RNA NSP12APO NSP12RNA

(b)

Y420

F442

S4

A443 K2

F429


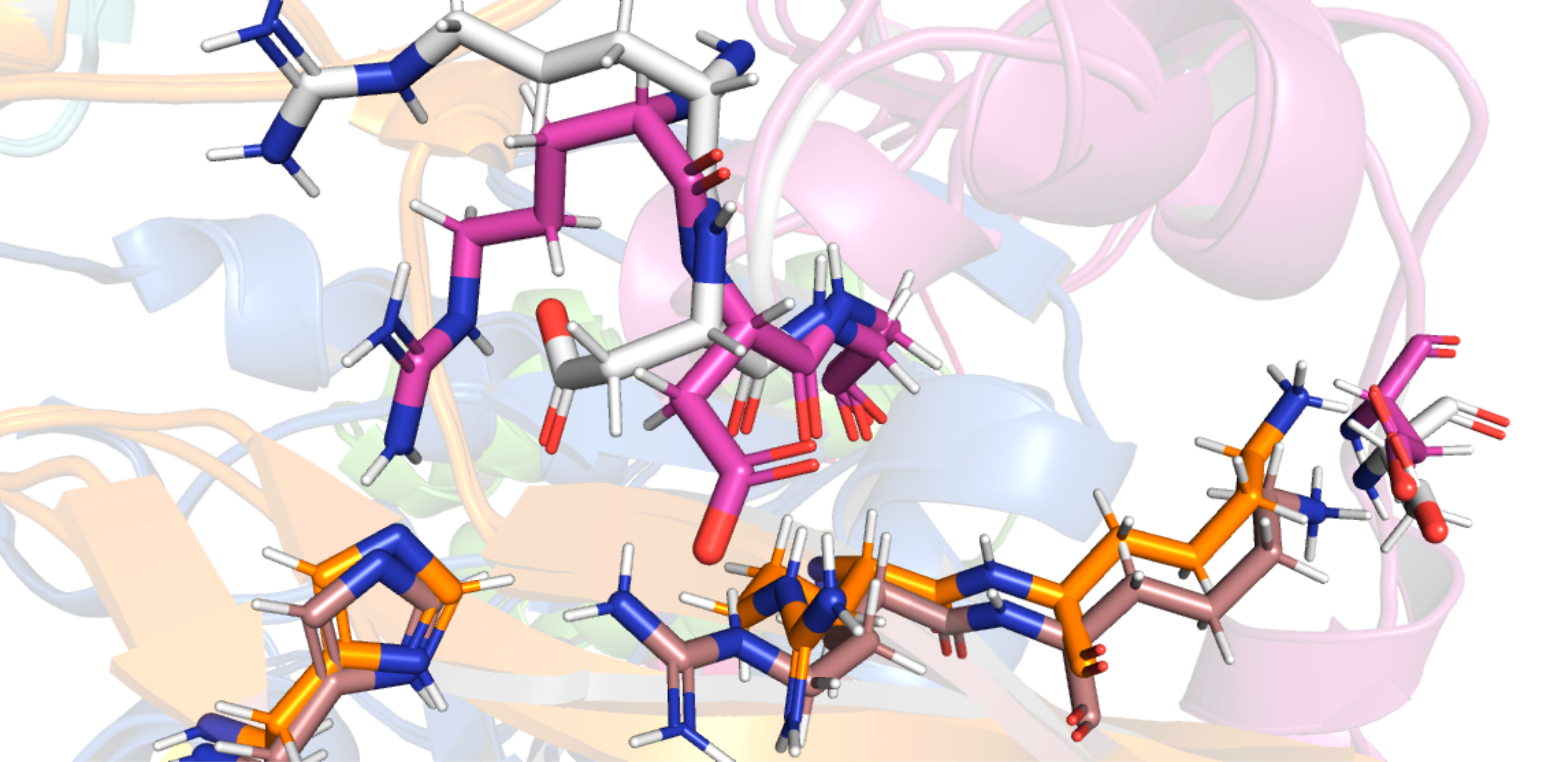


(c)

R111

D99

D112

K332

H355

R331

E431

**Figure S1.** Overlapped molecular structure of the amino acids in the complexes NSP12–NSP7*^APO^*,

NSP12–NSP7*^RNA^*, NSP12–NSP8*^APO^*, and NSP12–NSP8*^RNA^*. In the left side of the panel, there is a legend

1 1

representing the colors of the carbon atoms of the amino acids in each protein.

**Table S1.** Energy values (in kcal mol*−*1) of the most important residue-residue interaction pairs for the dimer NSP12–NSP8_1_.

1 1

| NSP12–NSP8_1_ | Energy (kcal.mol*−*1) | NSP12–NSP8_1_ | Energy (kcal.mol*−*1) |
| --- | --- | --- | --- |
| Residues | NSP12–NSP8*AP O* NSP12–NSP8*RNA* | Residues | NSP12–NSP8*AP O* NSP12–NSP8*RNA*  1 1 |
| L514–K79 | *−*3*.*43 *−*4*.*71 | V330–V115 | *−*1*.*99 *−*3*.*04 |
| D517–K79 | *−*4*.*42 *−*4*.*38 | L271–V115 | *−*2*.*12 *−*1*.*62 |
| R513–K79 | 0*.*48 *−*3*.*72 | L329–V115 | *−*4*.*40 *−*4*.*76 |
| D523–R80 | *−*1*.*22 *−*5*.*36 | P328–P116 | *−*2*.*98 *−*2*.*63 |
| F368–R80 | *−*2*.*54 *−*3*.*39 | L329–P116 | *−*3*.*00 *−*2*.*82 |
| S518–R80 | *−*2*.*79 *−*2*.*89 | V330–L117 | *−*2*.*97 *−*2*.*66 |
| W509–V83 | *−*2*.*12 *−*1*.*36 | A379–L117 | *−*2*.*01 *−*1*.*87 |
| L514–V83 | *−*1*.*59 *−*2*.*26 | P328–L117 | *−*3*.*09 *−*3*.*16 |
| F368–T84 | *−*1*.*78 *−*2*.*02 | T324–N118 | *−*2*.*73 *−*2*.*50 |
| W509–A86 | *−*3*.*04 *−*2*.*25 | F326–N118 | *−*2*.*29 *−*0*.*99 |
| L371–M87 | *−*2*.*21 *−*3*.*03 | A383–I120 | *−*2*.*02 *−*1*.*40 |
| W509–M87 | *−*2*.*98 *−*2*.*25 | L387–L122 | *−*2*.*02 *−*2*.*72 |
| L371–Q88 | *−*1*.*64 *−*2*.*35 | L387–K127 | *−*2*.*89 *−*3*.*71 |
| W509–M90 | *−*2*.*02 1*.*25 | N386–K127 | *−*0*.*78 *−*5*.*45 |
| S384–M94 | 5*.*33 *−*2*.*48 | L387–L128 | *−*5*.*62 *−*6*.*45 |
| P339–L95 | 0*.*38 2*.*39 | L387–M129 | *−*3*.*61 *−*3*.*75 |
| S384–K97 | *−*3*.*51 *−*5*.*71 | L388–M129 | *−*1*.*66 *−*5*.*71 |
| G385–K97 | *−*4*.*12 *−*2*.*60 | L389–M129 | *−*1*.*90 *−*3*.*85 |
| S384–L98 | *−*2*.*12 *−*0*.*89 | L389–V130 | *−*5*.*30 *−*6*.*53 |
| K332–D99 | *−*14*.*74 *−*11*.*63 | L389–V131 | *−*2*.*89 *−*3*.*56 |
| P339–D99 | 2*.*04 2*.*33 | D390–V131 | *−*3*.*26 *−*4*.*80 |
| K332–N104 | *−*6*.*45 *−*2*.*22 | K391–V131 | *−*2*.*57 *−*3*.*51 |
| K332–I107 | *−*2*.*08 *−*1*.*98 | R392–V131 | *−*3*.*02 *−*3*.*17 |
| H355–R111 | *−*5*.*77 *−*0*.*16 | K391–I132 | *−*1*.*35 *−*2*.*15 |
| D274–R111 | *−*1*.*68 *−*3*.*51 | R392–P133 | *−*1*.*94 *−*2*.*52 |
| R331–D112 | *−*8*.*16 *−*4*.*66 | K391–T137 | *−*2*.*60 *−*1*.*87 |
| R331–G113 | *−*4*.*80 *−*0*.*65 | L389–Y149 | *−*3*.*58 *−*2*.*69 |
| V330–C114 | *−*3*.*48 *−*2*.*46 | F407–A162 | *−*1*.*66 *−*2*.*29 |
| R331–C114 | *−*4*.*72 0*.*49 | V405–I185 | *−*2*.*45 *−*1*.*85 |
| Y273–C114 | *−*1*.*13 *−*2*.*77 |  |  |
